# Consistency analysis of two US techniques for evaluating hepatic steatosis in patients with metabolic dysfunction-associated steatotic liver disease

**DOI:** 10.1186/s12880-024-01549-1

**Published:** 2025-01-07

**Authors:** Fei Chen, Jingjing An, Long Deng, Jing Wang, Ruiling He

**Affiliations:** 1 Department of Ultrasound, The First Hospital of Lanzhou University, Lanzhou, 730000 China; 2 Department of Ultrasound, Donggang Branch the First Hospital of Lanzhou University, Lanzhou, 730000 China

**Keywords:** Metabolic dysfunction-associated steatotic liver disease, Liver steatosis, Hepatorenal index, Attenuation coefficient, Ultrasonography

## Abstract

**Background:**

US tools to quantify hepatic steatosis have recently been made clinically available by different manufacturers, but comparative data on their consistency are lacking.

**Objective:**

US tools to quantify hepatic steatosis have recently been made clinically available by different manufacturers, but comparative data on their consistency are lacking. The aim of our study was to compare the diagnostic consistency for evaluating hepatic steatosis by two different US techniques, hepatorenal index by B-mode Ratio and attenuation coefficient by attenuation imaging (ATI).

**Methods:**

Patients with suspicion or previously diagnosed of metabolic dysfunction-associated steatotic liver disease (MASLD) who attended fatty liver consulting room from June 2023 to September 2023 were prospectively recruited. Patients underwent two different US techniques of B-mode Ratio and ATI, and laboratory test were collected. According to previously proposed cut-off values, B-mode Ratio ≥ 1.22, 1.42, 1.54, and ATI ≥ 0.62, 0.70, and 0.78 dB/cm/MH were used for assessing of mild, moderate, and severe hepatic steatosis, respectively. Kappa consistency test was used to evaluate the consistency of hepatic steatosis.

**Results:**

A total of 62 patients were enrolled, including 44 males (71.0%) with an age of (41 ± 13) years and a body mass index of (27.0 ± 3.5) kg/m^2^. In the hyperlipidemia group, the B-mode Ratio and ATI were significantly higher than those in the non-hyperlipidemia group, with values of 1.68 ± 0.39 vs. 1.28 ± 0.35 (*p* = 0.001) and 0.74 ± 0.12 dB/cm/MH vs. 0.64 ± 0.11 dB/cm/MH (*p* = 0.005), respectively. The correlation coefficient between B-mode Ratio and ATI was 0.732 (*p* < 0.001). Using B-mode Ratio and ATI as diagnostic criteria for MASLD, the proportion of patients with MASLD was 79% and 82%, respectively. The Kappa coefficient for assessing MASLD was 0.90 (*p* < 0.001). Furthermore, these two different US techniques were used for grading hepatic steatosis, with no, mild, moderate, and severe steatosis accounting for 21%, 18%, 13%, and 48%, as well as 18%, 29%, 22%, and 31%, respectively. The linear weighted Kappa coefficient for staging hepatic steatosis was 0.78 (95% confidence interval: 0.68–0.87, *p* < 0.001).

**Conclusion:**

The non-invasive methods of two different US techniques based on B-mode Ratio and ATI have good consistency for evaluating hepatic steatosis, and can be used for large-scale community screening.

## Introduction

Nonalcoholic fatty liver disease (NAFLD) affects over 30% of the global population and is a common cause of chronic liver disease [1, 2]. The number of NAFLD patients is increasing with the rising prevalence of global obesity and metabolic syndrome [3]. At the European Association for the Study of the Liver in June 2023, it was proposed to rename NAFLD to “metabolic dysfunction-associated steatotic liver disease” (MASLD) to emphasize the importance of metabolic abnormalities and to avoid stigmatization [4]. MASLD is associated with an increased risk of type 2 diabetes and cardiovascular diseases, as well as metabolic dysfunction-associated steatohepatitis (MASH), liver cirrhosis, and hepatocellular carcinoma [5, 6]. Other chronic liver diseases also frequently coexist with hepatic steatosis, and the progression of chronic liver disease can be modified by liver lipids [7, 8]. Therefore, the detection and quantification of hepatic steatosis are crucial for the management of MASLD patients.

Although liver biopsy is considered as the reference method for the diagnosis of hepatic steatosis and fibrosis, it is impracticable for the screening or longitudinal follow-up at the population level because of its cost and risk related to this invasive procedure [9]. Non-invasive methods, magnetic resonance imaging (MRI)-proton density fat fraction as well as magnetic resonance elastography have emerged as the leading methods for assessing liver fat content and fibrosis in terms of accuracy, precision, and reproducibility [8, 10, 11]. However, like liver biopsy, MRI is expensive and time-consuming, and not routinely available in many centers. Currently, liver stiffness (LS) is the most widely used non-invasive method for assessing liver fibrosis stages in clinical practice [11].

US has many advantages, but the estimation of liver fat content has long been subjective with insufficient diagnostic performance, particularly in diagnosing mild steatosis for which sensitivity is only 60–65% [12, 13]. Several technologies have provided improved assessment of liver fat compared with conventional US by implementing quantitative approaches. The hepatorenal index is a promising US steatosis parameter, which have showed high accuracy for the diagnosis of any steatosis (≥ 5%) [14, 15]. The manufacturer of Supersonic Imagine developed the B-mode Ratio, which is based on the grayscale ratio of the liver and renal cortex, for the quantitative evaluation of the grade of liver steatosis [16, 17]. Moreover, the attenuation coefficient has been developed and commercialized by several vendors, quantifying liver fat by measuring the attenuation of radiofrequency, such as controlled attenuation parameter (CAP), ultrasound-guided attenuation parameter, and attenuation imaging (ATI) [8]. These measurements represented by ATI, are different from CAP in that they are incorporated with B-mode ultrasonography, which may result in more reliable and accurate measurements [18–20]. Our study aimed to compare the diagnostic consistency for evaluating hepatic steatosis by two different US techniques, hepatorenal index by B-mode Ratio and attenuation coefficient by ATI.

## Materials and methods

### Patients

From June 2023 to September 2023, we prospectively enrolled consecutive patients with suspicion or previously diagnosed of MASLD who attended fatty liver consulting room. These patients are over 18 years old, without significant alcohol consumption, without use of medications with steatogenic potential, and with no other etiology of chronic liver disease (viral hepatitis, autoimmune liver disease, and drug-induced liver disease). Significant alcohol consumption was defined as intake of 140–350 g in women and 210–420 g in men at weekly or an average daily 20–50 g in women and 30–60 g in men [21]. Additional exclusion criteria included the presence or history of decompensated cirrhosis (such as ascites, variceal bleeding, or hepatic encephalopathy), the presence or history of hepatocellular carcinoma, chronic kidney disease, and liver congestion owing to right-sided heart failure. For all patients, liver fat content was quantified the same day with two US techniques based on B-mode Ratio and ATI. Two US examinations were performed on each patient by trained and experienced sonographers (F.C. and R.H., to obtain B-mode Ratio and ATI, with more than 10 years of experience in liver US). B-mode Ratio was performed first, followed by ATI performed by another operator who was unaware of the result of the previous examination. Both operators randomized performed ultrasounds in both methods. This study was performed in accordance with the ethical guidelines of the Declaration of Helsinki and was approved by the institutional review board (LDYYLL-2024-483). Written informed consent was obtained from all patients.

### B-mode ratio

Ultrasound examination was carried out using Aixplorer ultrasound scanner (Supersonic Imagine, France) with B-mode Ratio software. A 1–6 MHz curved array convex probe was used.

Patients had fasted at least 6 h and were in the supine position with their right arms raised above their heads. The operator obtained the image that visualized both the right liver lobe and the right kidney in the same plane. On a frozen image, the measurement of the B-mode signal was taken by two regions of interests (ROIs), with the diameter of 6 mm, placed in the right liver parenchyma and right renal cortex. The positions of the ROIs should comply with the following requirements: both ROIs should be positioned at the same depth. They should avoid vascular and biliary structures for the liver, as well as hepatic or renal lesions. They should be far from artifacts, especially costal and pulmonary artifacts. The value of B-mode Ratio was directly calculated and provided by the device. Each patient underwent five measurements, and the median value was recorded.

### Liver stiffness measurement by two-dimensional shear wave elastography

Furthermore, we utilized the two-dimensional shear wave elastography (2D-SWE) feature of the Aixplorer ultrasound scanner for assessing LS.

LS was measured in the intercostal approach between the 7th and 9th ribs. The trapezoidal ROI, approximately 4×3-cm^2^, was placed in the parenchymal area devoid of large vessels and bile ducts, avoiding the noisy areas of rib artifacts. The Q-BOX was positioned at least 1 cm but not more than 6 cm from the liver capsule, with a diameter of no less than 1.5 cm. The value of LS was depicted in kPa. The latest European Federation of Societies for Ultrasound in Medicine and Biology guidelines were followed, the stiffness of the liver was measured five times and the median values was recorded [22]. The reliable value of LS was defined as the stability index of image ≥ 80% and the interquartile range/median ratio < 30%.

### ATI

The examination was performed using a Cannon Aplio i800 ultrasound system (Canon Medical Systems Corporation, Japan) and an ultra-wideband convex i8CX1 ultrasound probe.

Similar to the 2D-SWE measurement, a liver parenchyma region free of artifacts, vessels, and large biliary ducts was identified under B-mode guidance. Within the trapezoid sample box (height: 1.5–10 cm from the skin surface; width: approximately 6 cm), the operator placed an approximately 3×3- cm^2^ trapezoidal ROI with homogeneous color possibly, avoiding reverberation artifacts and large hepatic vessels, to obtain the value of ATI and record the value of R^2^. The R^2^ value represents the reliability of steatosis assessment, and a value ≥ 0.9 is considered excellent. Five ATI measurements were obtained, and the median value was documented.

### Laboratory tests

Blood samples were collected from all patients in a fasting state. Biochemical parameters, including serum aspartate aminotransferase; alanine aminotransferase; total cholesterol; triglycerides; low-density lipoprotein cholesterol; and high-density lipoprotein cholesterol were determined using a fully automated biochemical analyzer (Olympus AU400, Japan).

### Diagnostic criteria

According to Moret A et al. study, the cut-off values by B-mode Ratio of 1.22, 1.42, 1.54 were used to assess mild, moderate, and severe hepatic steatosis, respectively [17].

According to Jang JK et al. study, the cut-off values by ATI of 0.62, 0.70, 0.78 dB/cm/MH were used to grade mild, moderate, and severe hepatic steatosis, respectively [20].

LS by 2D-SWE great than 7 kPa was considered to have significant fibrosis [23].

According to the guideline for Lipid Management [24], the diagnostic criteria for dyslipidemia include elevated serum levels of cholesterol and/or triglycerides, which can be diagnosed by meeting one of the following five items. (1) plasma triglycerides ≥ 1.7 mmol/L; (2) plasma high-density lipoprotein cholesterol ≤ 1.0 mmol/L for man and ≤ 1.3 mmol/L for woman; (3) plasma low-density lipoprotein cholesterol ≥ 3.4 mmol/L; (4) plasma total cholesterol ≥ 5.2 mmol/L; (5) undergoing lipid lowering treatment.

### Statistical analyses

Continuous variables were expressed as mean ± standard deviation (SD) or median (interquartile range [IQR]) appropriately, and categorical variables as numbers (percentages). For group comparisons of continuous and categorical variables, Student’s *t-*test or Mann-Whitney test and Chi-square test were used, as appropriate. The consistency for assessing MALD and the grade of hepatic steatosis using two non-invasive methods, B-mode Ratio and ATI, was analyzed by Kappa coefficient and linear weighted Kappa coefficient. P values of < 0.05 were considered statistically significant. The data analysis was performed using SPSS (25.0.02) and GraphPad Prism (8.0.1).

## Results

### Patients characteristics

A total of 66 patients were enrolled, 2 subjects with failure of ATI; and 2 without laboratory data. Finally, our study included 62 patients [mean age, 41 ± 13 years; mean body mass index, 27.0 ± 3.5 kg/m²; 44 (71%) males]. Obesity was present in 39% of patients (24 of 62), and hyperlipidemia in 74% (46 of 62). All enrolled patients successfully underwent LS. The mean value of 2D-SWE LS was 5.6 ± 1.5 kPa. The baseline characteristics of enrolled patients are shown in Table 1.

**Table 1:** Patient characteristics

Variable	Overall	Non-hyperlipidemia group (*n*=16)	Hyperlipidemia group (*n*=46)	*P*
	(*n*=62)			
Male sex	44 (71.0)	12 (75.0)	32 (69.6)	0.926
Age (years)	41 ± 13	40 ± 12	42 ± 13	0.626
BMI (kg/cm^2^)				
Mean	27.0 ± 3.5	26.1 ± 2.1	27.3 ± 3.8	0.127
< 24	8 (12.9)	1 (6.3)	7 (15.2)	
24-27.9	30 (48.4)	12 (75.0)	18 (39.1)	
≥ 28	24 (38.7)	3 (18.8)	21 (45.7)	
AST (U/L)	24.0 (18.5)	20.8 (10.5)	26.4 (33.3)	0.107
ALT (U/L)	33.0 (40.9)	21.5 (19.1)	41.5 (60.8)	0.053
TC (mmol/L)	4.52 ± 0.97	3.97 ± 0.64	4.72 ± 0.99	0.011
TG (mmol/L)	2.10 ± 1.24	1.20 ± 0.33	2.42 ± 1.29	<0.001
LDL-C (mmol/L)	2.94 ± 0.69	2.55 ± 0.56	3.08 ± 0.68	0.012
HDL-C (mmol/L)	1.09 ± 0.22	1.15 ± 0.12	1.07 ± 0.25	0.225
B-mode Ratio	1.57 ± 0.42	1.28 ± 0.35	1.68 ± 0.39	0.001
ATI (dB/cm/MHz)	0.72 ± 0.13	0.64 ± 0.11	0.74 ± 0.12	0.005
2D-SWE LS (kPa)	5.6 ± 1.5	4.9 ± 0.9	5.9 ± 1.5	0.022

Note: Values were presented as mean ± SD when continuous variables had normal distribution, or by median (IQR) for non-normal distribution. Categorical data were presented as frequencies (%)

BMI = body mass index; AST = aspartate aminotransferase; ALT = alanine aminotransferase; TC = total cholesterol; TG = triglycerides; LDL-C = low-density lipoprotein cholesterol; HDL-C = high-density lipoprotein cholesterol; ATI = attenuation imaging; 2D-SWE LS = two dimensional-shear wave elastography liver stiffness

13% (8/62) of patients with 2D-SWE LS ≥ 7 kPa have progressed to MASH. Among these 8 patients with MASH, 2 had normal weight, 3 were overweight (body mass index ≥ 24 kg/m²), and 3 were obese(body mass index ≥ 28 kg/m²), and all patients had hyperlipidemia. The baseline characteristics of patients with MASH is shown in Table 2.

**Table 2:** Patient characteristics in MASH

Variable	MASH group (*n*=8)
Male sex	5 (62.5)
Age (years)	49 ± 15
BMI (kg/cm^2^)	
Mean	27.4 ± 5.1
< 24	2 (25.0)
24-27.9	3 (37.5)
≥ 28	3 (37.5)
AST (U/L)	44.7 (52.0)
ALT (U/L)	73.1 (97.5)
TC (mmol/L)	4.41 ± 0.99
TG (mmol/L)	3.39 ± 1.78
LDL-C (mmol/L)	2.88 ± 0.81
HDL-C (mmol/L)	0.95 ± 0.12
B-mode Ratio	1.96 ± 0.24
ATI (dB/cm/MHz)	0.81 ± 0.11
2D-SWE LS (kPa)	8.5 ± 1.3

Note: Values were presented as mean ± SD when continuous variables had normal distribution, or by median (IQR) for non-normal distribution. Categorical data were presented as frequencies (%)

MASH = metabolic dysfunction-associated steatohepatitis; BMI = body mass index; AST = aspartate aminotransferase; ALT = alanine aminotransferase; TC = total cholesterol; TG = triglycerides; LDL-C = low-density lipoprotein cholesterol; HDL-C = high-density lipoprotein cholesterol; ATI = attenuation imaging; 2D-SWE LS = two dimensional-shear wave elastography liver stiffness

### Distribution of B-mode ratio and ATI in hyperlipidemia and non-hyperlipidemia groups

**Fig. 1:**
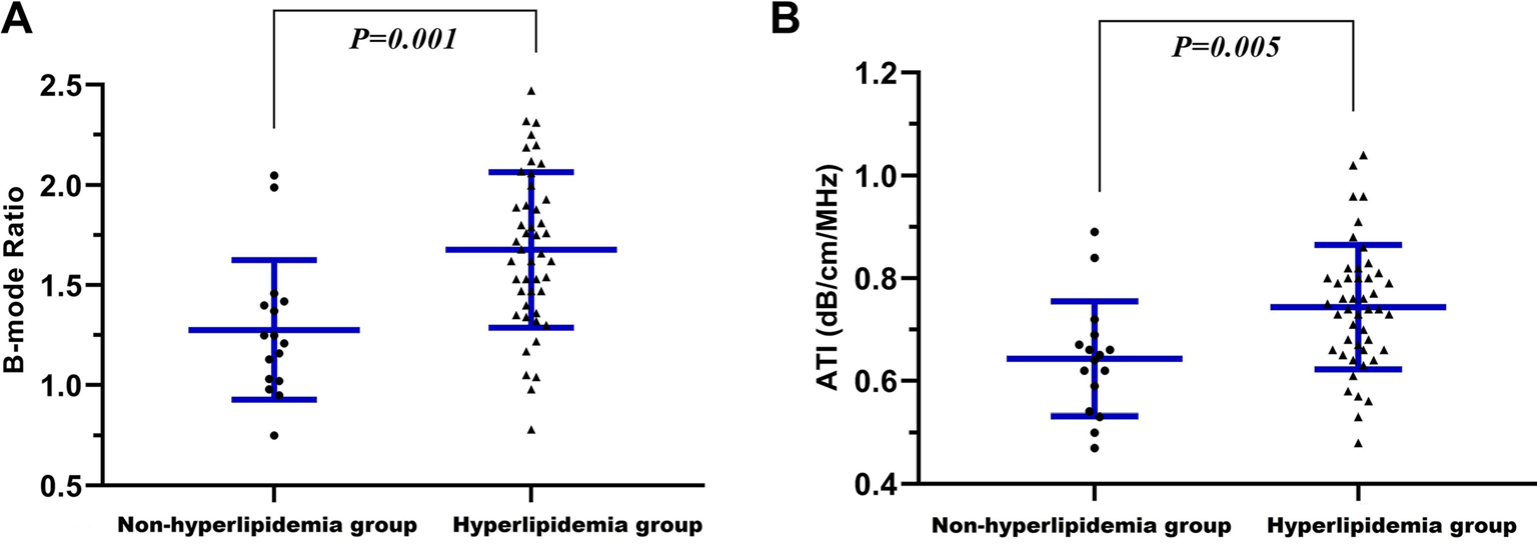
Distribution of B-mode Ratio (**A**) and ATI (**B**) in hyperlipidemia and non-hyperlipidemia groups. ATI, attenuation imaging

The mean (± SD) values for B-mode Ratio and ATI were 1.57 ± 0.42 and 0.72 ± 0.13 dB/cm/MHz, respectively (Table 1). The mean (± SD) B-mode Ratio for subjects with hyperlipidemia and non-hyperlipidemia were 1.68 ± 0.39 and 1.28 ± 0.35, and B-mode Ratio with hyperlipidemia group were significantly higher than non-hyperlipidemia group (*p* = 0.001) (Fig. 1A). Additionally, the ATI for hyperlipidemia group were significantly higher than that for non-hyperlipidemia group (*p* = 0.005), with a mean (± SD) of 0.74 ± 0.12 dB/cm/MHz and 0.64 ± 0.11 dB/cm/MHz, respectively (Fig. 1B).

**Fig. 2:**
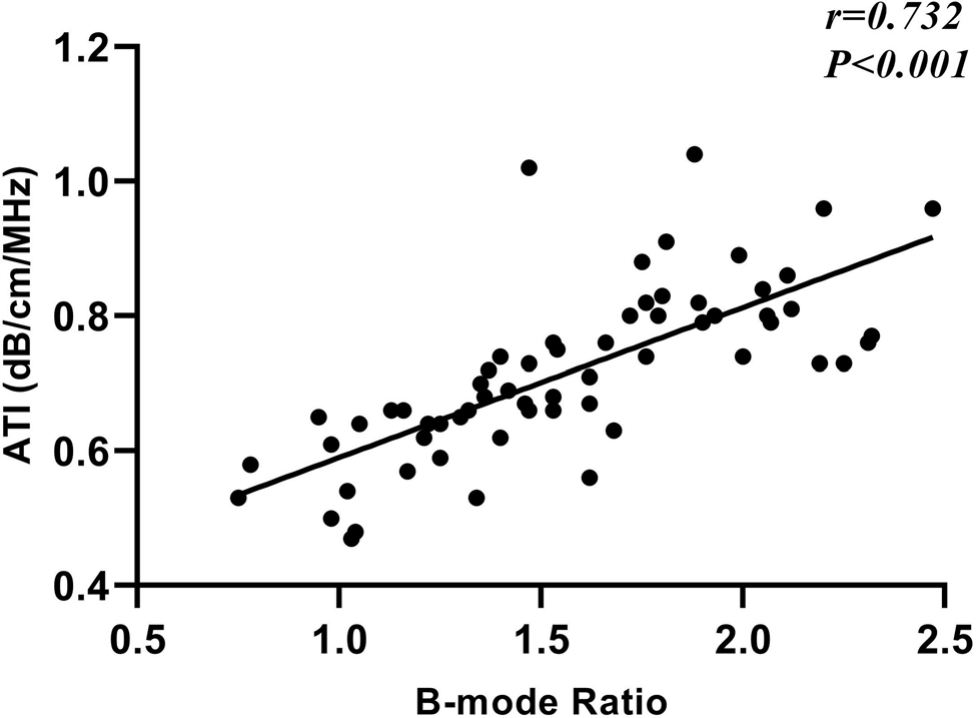
Scatterplot of B-mode Ratio and ATI values. ATI, attenuation imagin

Pearson correlation analysis showed a strong positive correlation between B-mode Ratio and ATI, *r* = 0.732, *p* < 0.001 (Fig. 2).

### Kappa consistency test for MASLD

**Fig. 3:**
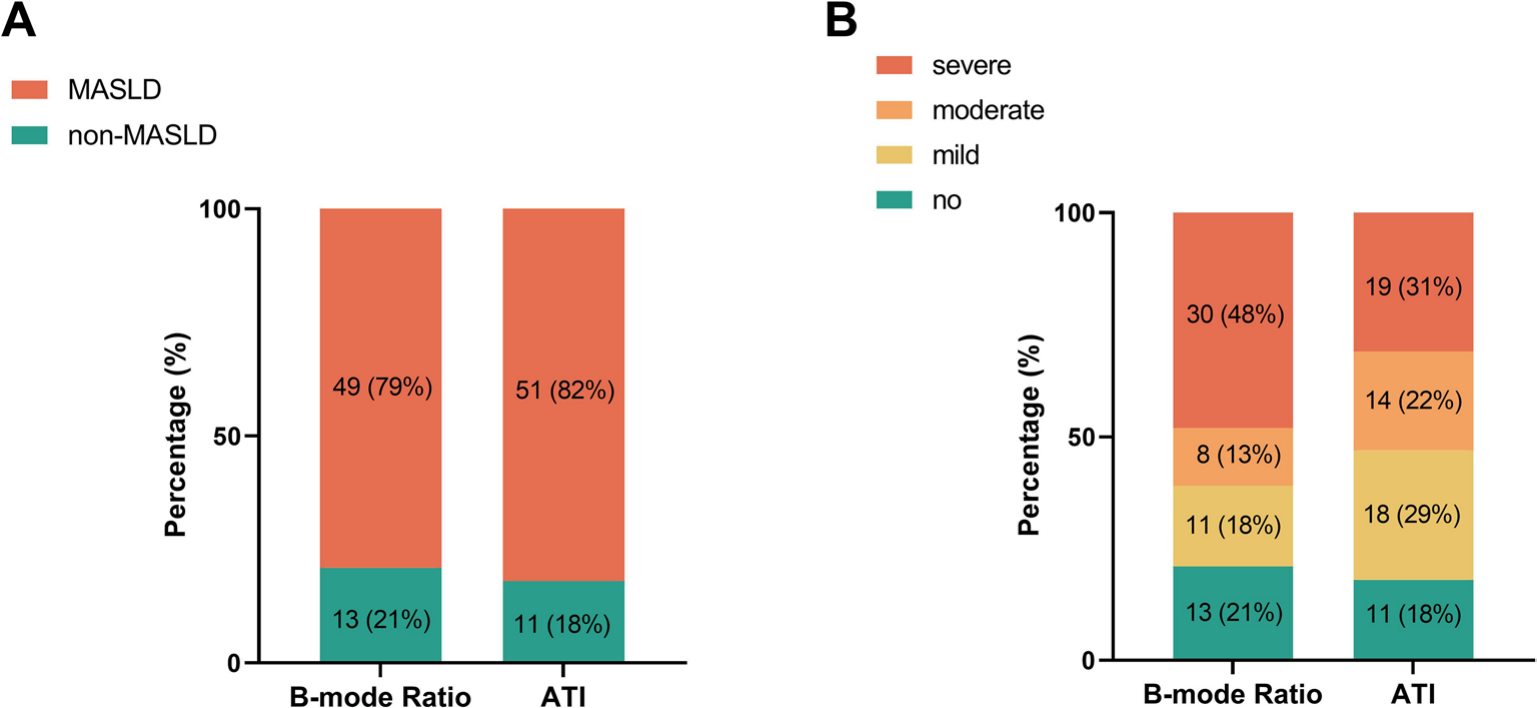
Numbers and proportions of patients with MASLD (**A**) and the grade of hepatic steatosis (**B**) by two different US techniques. MASLD, metabolic dysfunction-associated steatotic liver disease. ATI, attenuation imaging

Using B-mode Ratio ≥ 1.22 and ATI ≥ 0.62 dB/cm/MHz as diagnostic criteria for MASLD, the proportion of patients with MASLD was 79% and 82%, respectively (Fig. 3A).

The Kappa consistency test showed that these two non-invasive methods yielded consistent classification for 60 patients, with only 2 patients indicating inconsistency. These two subjects were identified as MASLD by ATI, while as no MASLD by B-mode Ratio. Overall, the Kappa coefficient for the assessing of MASLD was 0.90 (*p* < 0.001), indicating excellent consistency.

### Weighted kappa consistency test for grading hepatic steatosis

Applying B-mode Ratio values of 1.22, 1.42, and 1.54 as diagnostic criteria for mild, moderate, and severe hepatic steatosis, the proportions of patients with no, mild, moderate, and severe steatosis were 21%, 18%, 13%, and 48%, respectively. Applying ATI values of 0.62, 0.70, and 0.78 dB/cm/MHz as diagnostic criteria, the proportions of subjects with no, mild, moderate, and severe steatosis were 18%, 29%, 22%, and 31%, respectively (Fig. 3B).

The linear weighted Kappa coefficient showed that these two methods yielded consistent classification for 44 subjects and inconsistent classification for 18 subjects. Of them, 11 cases identified as severe by B-mode Ratio while moderate by ATI; 5 cases identified as moderate by B-mode Ratio while mild by ATI; 2 cases identified as no hepatic steatosis by B-mode Ratio while mild by ATI. Overall, the linearly weighted Kappa coefficient for the grade of hepatic steatosis using these two non-invasive methods was 0.78 (95% CI: 0.68–0.87, *p* < 0.001), showing good consistency.

## Discussion

We conducted a prospective study including adults with suspected or previously diagnosed MASLD, using B-mode Ratio and ATI to quantify liver fat content. Our study aimed to investigate the diagnostic consistency of these two non-invasive US techniques for evaluating MASLD and the grade of hepatic steatosis. We observed good correlation between the B-mode Ratio and ATI (*r* = 0.732, *p* < 0.001). In addition, these two non-invasive methods showed excellent consistency for MASLD (Kappa coefficient of 0.90, *p* < 0.001) and good consistency for the grade of steatosis (Linearly Weighted Kappa coefficient of 0.78, *p* < 0.001).

The cut-off values for diagnosis of steatosis with mild, moderate, and severe based on B-mode Ratio and ATI vary significantly among original studies [16, 17, 20, 25–27]. The most likely cause of the heterogeneity in cut-off values is the different etiologies of chronic liver disease. Another factor is the differing standards for diagnosing hepatic steatosis, with some studies using liver biopsy and others using MRI-proton density fat fraction. Hence, the threshold of our study for hepatic steatosis was referenced from relatively large-sample original studies in which all or mostly patients suspected having MASLD, as well as the histological grade of steatosis was considered as the gold standard. In a prospective multicenter study by Moret A et al., 250 patients who underwent liver biopsy were recruited, and they found that the area under the receiver operating curves (AUROCs) of B-mode Ratio for ≥ S1, ≥ S2, and S3 were 0.90, 0.78, and 0.73, respectively, with optimal cut-off values of 1.22, 1.42, and 1.54 [17]. In another prospective study by Jang JK et al., 132 suspected MASH patients were included, using the histological grade of steatosis as a reference, the AUROCs of ATI for ≥ S1, ≥ S2, and S3 were all 0.92, with optimal cut-off values of 0.62, 0.70, and 0.78, respectively [20]. In our study, we found excellent consistency between B-mode Ratio and ATI in diagnosing MASLD using the cut-off values from aforementioned studies. Furthermore, when it comes to the grade of hepatic steatosis, we found that in cases of diagnostic inconsistency, B-mode Ratio tended to overestimate the grade of steatosis compared to ATI. Among 18 cases, B-mode Ratio overestimated one grade in 16 cases, while ATI overestimated one grade in only 2 cases. Overall, the two US techniques showed good consistency in the grade of steatosis.

US tools have recently emerged to quantify liver fat content, including B-mode Ratio based on the grayscale ratio of the liver and renal cortex, and attenuation techniques based on the quantitative measurement of the energy lost by the ultrasound wave when passing through a medium [8]. As a simple ratio of signal, the B-mode Ratio is a basic technology that can be easily performed even on JPEG images, as shown by Marshall et al. [14]. Although studies have shown that B-mode Ratio has relatively lower diagnostic performance for moderate and severe steatosis compared to ATI, it does not require any post-processing on US techniques and can be directly calculated and given through software [14, 17, 18]. Therefore, it opens the possibility to large-scale community screening of steatosis. It assists primary care, especially community physicians, to identify patients who should or should not be referred to specialists for further examination regarding the clinical situation. Moreover, in other clinical situations requiring knowing whether or not steatosis is present, but not a precise quantification (such as follow-up of diet, and lifestyle advice, etc.), the B-mode Ratio appears to be a sufficient, accessible, and low-cost tool. Currently, it can only be used with machine of specific manufacturer, as it is the only one equipped with software to calculate this ratio. Given the simplicity of B-mode Ratio measurement, other manufacturers could install calculation software in existing US machines in primary care and community hospitals, facilitating large-scale steatosis screening economically.

Unlike hepatorenal index, attenuation techniques require post-processing with US machines to quantify liver fat by measuring the attenuation of radiofrequency signals. CAP is the first approved method for the quantification of liver fat based on attenuation evaluation, and is widely used to assess hepatic steatosis [8, 19]. However, its diagnostic performance remains limited (particularly in a steatosis grade of S2 or higher), and the optimal cut-offs value varies across etiologies [19, 28]. Several US manufacturers have developed their own proprietary attenuation imaging techniques. A recent meta-analysis compiled the results of 11 studies showed satisfactory diagnostic performance for the US attenuation coefficient, with an AUC of 0.83 for diagnosis of steatosis grade S1 and higher and 0.91 for diagnosis of grade S2 and higher [18]. The advantage of ATI is that it allows the use of B-mode image to guide the selection of the measurement area, offering comparable diagnostic accuracy to CAP with a lower measurement failure rate [8, 29].

MASLD patients typically have an insidious onset, and the progression of liver disease is slowly. For patients with MASH, liver fibrosis progresses by one grade on average every 7–10 years. Bridging fibrosis and cirrhosis are independent predictors of adverse liver outcomes. Over a 10–20 year follow-up period, the incidence of cirrhosis in MASLD patients is only 0.6-3%; in contrast, the incidence of cirrhosis in MASH patients is as high as 15-25% within 10–15 years [30]. Hence, MASH has an important clinical implication in treatment because it is possible to reverse MASH before the development of cirrhosis or hepatocellular carcinoma if optimal intervention is provided [31]. In our study, 13% (8/62) of patients were diagnosed with MASH (2D-SWE LS ≥ 7 kPa). Among these 8 cases, 75% (6/8) were overweight or obese, and all patients had hyperlipidemia. These patients are advised to undergo specialist examinations and receive individualized treatment to prevent liver-related adverse outcomes and cardiovascular disease.

The main limitation of our study is the lack of liver biopsies or MRI-proton density fat fraction. Therefore, the diagnostic accuracy of B-mode Ratio or ATI could not be assessed in this study. On the other hand, our results reflect real-life clinical practice, in which the number of liver biopsies has been steadily decreasing due to the abundance of new and established non-invasive methods. Quantitative assessment of liver fat content using different US techniques is more suitable for clinical scenarios. Therefore, it is particularly important to explore the consistency of different US techniques in assessing hepatic steatosis, highlighting the primary objective of our research. Secondly, this study was a single-center study and had a small sample of patients. Next, we plan to conduct a large-sample multicenter study to investigate the diagnostic accuracy of B-mode Ratio and ATI using liver biopsy or MRI-proton density fat score as the standard. Furthermore, as the B-mode Ratio is derived from the liver and renal cortical grayscale ratio, it could be influenced by chronic kidney disease, which can alter the renal cortex attenuation in ultrasound. It would be interesting to evaluate the relationship between renal function and B-mode Ratio value. Finally, for MASH patients undergoing individualized treatment, whether non-invasive methods can effectively assess their therapeutic response remains to be further studied.

In conclusion, the non-invasive methods of two different US techniques based on B-mode Ratio and ATI have good consistency for evaluating hepatic steatosis, and can be used for large-scale community screening.

## Data Availability

All data generated or analyzed during this study are included in this published article.
